# From principles to practice: operationalizing responsible AI governance across predictive and generative clinical AI

**DOI:** 10.3389/fdgth.2026.1965076

**Published:** 2026-10-27

**Authors:** Chung-Feng Liu, Chia-Jung Chen, Yen-Ling Ko

**Affiliations:** 1 Department of Medical Research, Chi Mei Medical Center, Tainan, Taiwan; 2 Department of Information Systems, Chi Mei Medical Center, Tainan, Taiwan; 3 Department of Internal Medicine, Chi Mei Medical Center, Tainan, Taiwan; 4 General Education Center, Chia Nan University of Pharmacy and Science, Tainan, Taiwan

**Keywords:** clinical artificial intelligence, clinical workflow, digital health, explainability, generative AI, human-AI interaction, predictive AI, responsible AI governance

## Abstract

Responsible artificial intelligence (AI) governance in healthcare is increasingly supported by international principles addressing transparency, accountability, safety, explainability, and lifecycle risk management. Yet a persistent implementation gap remains between these institution-level principles and the concrete controls encountered when an AI service is used in clinical practice. This Perspective argues that responsible AI should be operationalized at the functional points where clinical AI outputs are produced, explained, and communicated. We use three functional domains—Capability, Explainability, and Communication—as an organizing structure for translating existing governance principles into application-level specifications. The approach accommodates both predictive AI (PAI) and generative AI (GAI) while recognizing their different risk profiles. For PAI, governance emphasizes target and prediction-horizon definition, calibration, thresholds, drift, external validation, and alert burden. For GAI, it emphasizes task boundaries, source traceability, hallucination control, version management, human verification, and constraints on write-back or autonomous action. Two dialysis-related applications illustrate how the same governance vocabulary can be instantiated differently across AI modalities. Operationalizing responsible AI in this way does not introduce new governance principles; rather, it organizes existing requirements into observable, reviewable, and auditable controls embedded in clinical AI services and workflows.

## 1 Introduction

Clinical artificial intelligence (AI) is moving beyond standalone model development toward services embedded in electronic health records, monitoring systems, and clinical workflows. As this transition occurs, the central governance question is no longer limited to whether a model achieves acceptable discrimination or accuracy. Healthcare organizations must also determine what an AI output means, when it is applicable, how it should be interpreted, how it reaches the user, what actions it may trigger, and how the service is monitored and changed over time (1, 2).

International guidance has established a strong normative foundation for trustworthy and responsible AI. The World Health Organization (WHO) emphasizes autonomy, transparency, accountability, inclusiveness, and sustainability (1). The NIST AI Risk Management Framework addresses validity, reliability, safety, transparency, explainability, privacy, and ongoing risk management (2), while the NIST Generative AI Profile extends this perspective to risks that are particularly salient for generative systems (3). Healthcare-specific literature has further examined the applications and technical, organizational, and regulatory challenges associated with GAI in healthcare (4). At the health-system level, multidisciplinary governance approaches have also been developed to support the safe, equitable, and continuously monitored deployment of clinical AI (5). Regulatory guidance emphasizes transparency for machine-learning-enabled medical devices (6). SPIRIT-AI and CONSORT-AI extend protocol and trial-reporting guidance to AI interventions (7, 8), while DECIDE-AI addresses reporting for early-stage clinical evaluation of AI-driven decision-support systems (9).

However, principles do not implement themselves. A hospital may endorse transparency and human oversight while still lacking an operational answer to questions such as: Which model threshold should be disclosed? When should a prediction be suppressed because data are incomplete? How should a SHAP explanation be described so that it is not interpreted causally? Which source should accompany a generated statement? At what point must a clinician review, edit, confirm, or override an AI output? These are application-level questions, and they occur at different functional points in the service.

Prior explainable clinical decision support systems already combine model inference, explanation, and user-facing presentation. For example, explainable CDSS work has integrated inference with interpretable output and graphical interfaces (10), while human-centered XAI research distinguishes the extraction of explanations from the design of explanation interfaces (11). These contributions make clear that Capability–Explainability–Communication should not be presented as a novel technical pipeline. The unresolved opportunity is instead to use these familiar functional points as governance control points: locations at which responsible AI requirements can be translated into concrete specifications, disclosures, monitoring indicators, and human-use constraints.

This Perspective therefore proposes a shift from principle-based governance toward application-level operationalization. We use three functional domains—Capability, Explainability, and Communication—to organize responsible AI requirements across predictive AI (PAI) and generative AI (GAI). The contribution lies not in proposing new governance principles, but in organizing existing requirements into application-level specifications that define what an AI service is designed to do and under what performance and control requirements, what users need to understand and verify its results, and how those results enter clinical workflow and are acted upon and recorded.

## 2 Shared governance, differentiated risks

PAI and GAI can coexist within the same healthcare organization and even within the same clinical workflow, but they should not be governed as if their outputs and failure modes were equivalent. PAI typically produces probabilities, classifications, or risk strata. Its governance therefore depends heavily on target definition, prediction horizon, calibration, threshold selection, subgroup performance, external validity, drift, and the clinical burden created by alerts (12). A model with acceptable area under the receiver operating characteristic curve can still be unsafe if its threshold is poorly matched to prevalence, if calibration deteriorates after deployment, or if excessive alerts are ignored.

GAI produces task-oriented content such as summaries, draft notes, reminders, retrieved knowledge, or conversational responses. GAI introduces additional risks related to hallucination, prompt sensitivity, provenance, and the reliability of generated content (3, 13). Recent healthcare-specific literature has further highlighted safety and security risks associated with clinical LLM use (14), hallucination and corresponding mitigation strategies (15), and broader technical, organizational, and regulatory challenges associated with the integration of GAI into healthcare (4). Healthcare-specific evaluation approaches have also incorporated clinician-in-the-loop assessment of dimensions such as correctness, completeness, helpfulness, and potential clinical harm (16).

Despite these differences, PAI and GAI share governance needs: clearly bounded intended use, version control, transparency, human review appropriate to risk, monitoring after deployment, incident handling, auditability, and mechanisms for change or withdrawal. The practical challenge is therefore to create a common governance language without forcing the two modalities into identical controls. A useful framework should make both the common foundation and the modality-specific requirements visible.

## 3 Three functional domains for operationalizing responsible AI

Figure 1 illustrates the three functional domains along the clinical AI information flow and their associated governance control items. These domains organize, rather than replace, the broader governance areas discussed above. Capability encompasses service scope and outputs together with performance, monitoring, version, and change-control requirements; Explainability addresses the information needed to understand and verify AI results; and Communication addresses human interaction with those results in clinical workflow. Lifecycle governance operates across the service, with model or system change and revalidation specified principally through Capability and broader lifecycle controls applied across all three domains.

**Figure 1:**
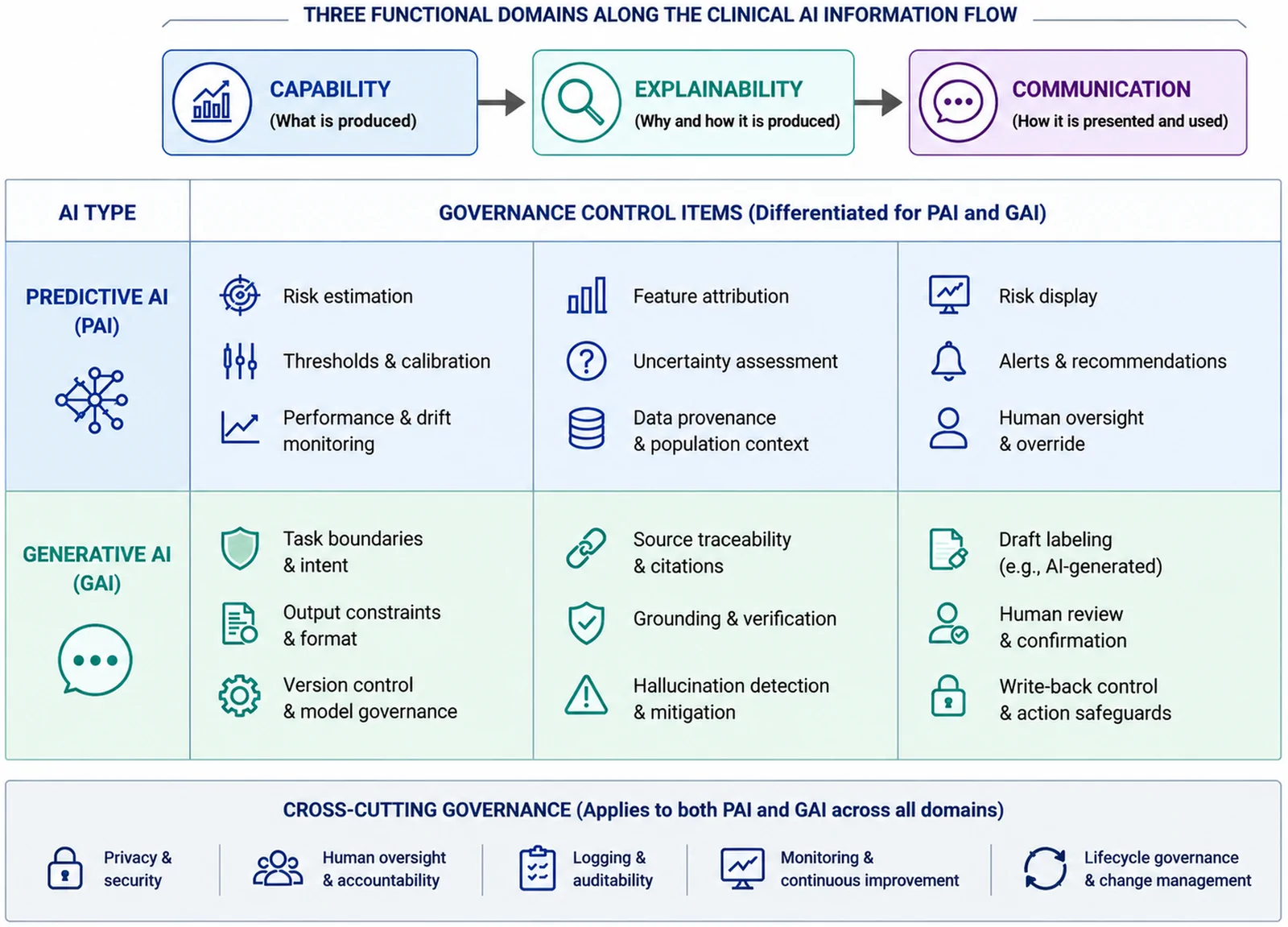
Functional domains along the clinical AI information flow and associated governance control items.

### 3.1 Capability: defining scope, performance, and control

The Capability domain concerns what an AI service is designed to do, under what conditions, and with what performance and control requirements. For PAI, this includes the target event, prediction horizon, eligible population, input variables, output scale, threshold strategy, calibration, update frequency, model version, and revalidation rules. Performance disclosure should extend beyond discrimination to indicators relevant to use, including sensitivity, positive predictive value, calibration, external validation, subgroup performance, and alert burden when applicable. Post-deployment monitoring should specify what constitutes drift or unacceptable degradation and what action follows detection (2, 6). For regulated AI-enabled devices, version management and revalidation should also consider applicable regulatory change-management mechanisms, such as the US FDA's Predetermined Change Control Plan (PCCP) guidance for prospective management of planned modifications (17).

For GAI, capability is defined by the intended task, such as generating a draft progress note or reminder. The service should specify prohibited outputs and actions, quality indicators, subgroup or equity evaluation where clinically relevant, model and prompt versions, and the conditions under which generation should be refused or escalated. Evaluation should be aligned with the intended clinical task and include clinically relevant dimensions of output quality and potential harm (16). A draft note is fundamentally different from a signed medical record entry; a retrieved SOP excerpt is different from a medical order. These boundaries should be explicit in both system documentation and user-facing design.

### 3.2 Explainability: making outputs understandable and verifiable

The Explainability domain addresses what information is required for a user to understand, contextualize, verify, and appropriately limit reliance on an AI output. Explainability in healthcare is not a single technical property; it has clinical, ethical, and legal dimensions (18). For PAI, local feature attribution, clinical trends, confidence information, error patterns, and subgroup performance can support interpretation. When SHAP is used, the interface and documentation should make clear that feature attribution describes model behavior rather than causal relationships (19). Missing data, out-of-scope inputs, and uncertainty should be visible rather than hidden behind apparently precise outputs.

For GAI, explainability is often better operationalized as traceability and verifiability than as feature attribution. Generated statements should be linked, where feasible, to identifiable clinical sources, timestamps, retrieved documents, or evidence markers. Retrieved content should be distinguishable from model-generated synthesis. When the available information is insufficient, refusal or explicit requests for human supplementation may be safer than fluent completion. This distinction is important because a persuasive generated narrative can appear clinically coherent even when its factual basis is incomplete.

### 3.3 Communication: governing how AI enters clinical work

The Communication domain concerns how outputs are presented, timed, reviewed, acted upon, and recorded within clinical work. This domain should not be reduced to graphical user-interface design. Clinical decision support is more likely to influence practice when it is integrated into workflow and delivered at an appropriate time and place (20), while poorly designed alerts can create burden and undermine adoption (21). For high-risk AI systems within its scope, the EU AI Act further requires effective human oversight, reinforcing the need for application-level controls that define oversight responsibilities and conditions for human intervention (22).

For PAI, communication controls include risk stratification displays, alert timing, escalation rules, links to SOPs, and the ability to acknowledge or override an alert. For GAI, communication controls include review-edit-confirm workflows, clear draft labeling, restrictions on automatic write-back, and preservation of user modifications. Importantly, governance must specify the user and the authority attached to each interaction: who receives the output, who may act, who may override, and which action is recorded. Depending on the type of AI result and its intended clinical use, these role-and-authority specifications should also define who is responsible for reviewing or verifying the result, whether human confirmation is required before action or documentation, and what actions, overrides, or modifications should be retained in the clinical record or audit trail. These questions become especially important when clinical work is distributed across physicians, nurses, pharmacists, technicians, and other team members. Human oversight and accountability therefore operate across the service, while the Communication domain is the point at which they become visible in actual use.

Communication is related to, but distinct from, Explainability. Explainability specifies what information users need to understand and verify an AI result and appropriately limit reliance on it, whereas Communication specifies how that result enters clinical workflow and who has authority and responsibility for subsequent action and documentation. Although some controls, such as traceability, may support both domains, the domains remain distinct according to their primary governance function.

## 4 From principles to application-level specifications

Operationalization requires a traceable connection between high-level governance expectations and concrete service controls. Table 1 translates the three functional domains into application-level specifications for PAI and GAI. Table 2 then situates these specifications against their nearest counterparts in established AI governance frameworks and clinical AI guidance. The substantial overlap is intentional: most underlying governance requirements are not unique to this framework. Rather, the three-domain approach organizes them into reviewable controls at the level of an individual clinical AI service: capability scope, performance, and control requirements; verification requirements; and workflow authority and record boundaries. Together, the two tables illustrate the level of specificity required for responsible AI requirements to become reviewable at the application level.

**Table 1:** Application-level governance specifications across the three functional domains for predictive and generative AI.

Functional domain	Governance question	Predictive AI (PAI)	Generative AI (GAI)
Capability	What is the service designed to do, for whom, under what conditions, and how are performance and change controlled?	Target and horizon; population and exclusions; thresholds; calibration; external/subgroup validation; drift; model version; revalidation; threshold-dependent performance and alert burden where clinically relevant.	Task and output type; prohibited outputs/actions; quality indicators; subgroup/equity evaluation where clinically relevant; model/prompt version; refusal conditions; output/version records.
Explainability	What must users know to understand, verify, and appropriately limit reliance on the output?	Feature attribution; clinical context and trends; uncertainty; missing/out-of-scope data; explanation limitations; non-causal interpretation.	Source attribution; timestamps; citation markers; retrieved-versus-generated distinction; factual verification; insufficiency/refusal mechanisms.
Communication	How does the output enter workflow, and how is human use controlled and recorded?	Risk display; alert timing and escalation; SOP linkage; acknowledgement; override; action and audit logs.	Draft labeling; review-edit-confirm; write-back restrictions; user modifications; human verification; audit trail.

**Table 2:** Crosswalk of the three functional domains with existing AI governance frameworks and clinical AI guidance.

Domain	Counterparts in existing frameworks and guidance	Application-level operationalization
Capability	Intended purpose and use; users and affected populations; application scope; inputs and outputs; performance and limitations; monitoring and change management (2, 7–9).	Capability specification: defines what each AI service is designed to do, for whom, and under what conditions, and links its intended scope to service-specific performance, version, monitoring, and revalidation controls.
Explainability	Transparency and documentation; explainability/interpretability; uncertainty and limitations; information supporting understanding and verification of outputs; human–AI interaction and error/failure analysis (2, 3, 7–9).	Verification requirement: defines what information must accompany each output for users to understand its basis and limitations, verify it when needed, and appropriately limit reliance; for GAI, this includes source provenance and distinction between retrieved and generated content.
Communication	Human oversight; roles and responsibilities; intended users and expertise; output presentation and use; workflow integration; human–AI interaction and documentation (2, 7–9).	Workflow authority and record boundary: defines who receives and verifies each output, who may act on or override it, when human confirmation is required, what may enter the clinical record, and which actions or modifications must be retained.

The second column summarizes the nearest conceptual counterparts to the specifications in Table 1 and does not imply one-to-one equivalence. The third column shows how these overlapping requirements are organized into reviewable controls at the level of an individual clinical AI service.

Several requirements cut across all three domains. Data access, interoperability, privacy, cybersecurity, identity and role management, logging, incident response, and lifecycle governance should not be treated as a separate downstream component; they are cross-cutting enabling controls that must be embedded wherever data and outputs move through the service. Standards-based integration with hospital information systems may be implemented through local or interoperable mechanisms according to institutional context. The governance perspective is therefore deliberately distinct from a software architecture specification: it identifies what must be controlled and disclosed, while the technical implementation may vary across organizations.

## 5 Illustrative clinical applications

Two dialysis-related AI services at Chi Mei Medical Center illustrate how the same three functional domains can be operationalized differently according to AI type. The predictive AI service for intradialytic hypotension (IDH) and its performance findings have been described in previously published work (23). The generative AI-assisted dialysis rounding support service has been implemented in clinical practice since April 2024. The GAI service supports physicians during dialysis rounds without replacing clinical judgment and has not undergone prospective clinical validation. Both examples illustrate governance requirements rather than validate the framework.

For the IDH predictive service, the Capability domain defines the target event, prediction horizon, eligible population, threshold strategy, and ongoing monitoring. In the IDH service, the target event was intradialytic systolic blood pressure <90 mmHg within the next 60 min in adults receiving maintenance hemodialysis; records lacking required clinical or laboratory information were excluded. The model operating threshold was 0.35511, selected to balance sensitivity and specificity. For user-facing presentation, model outputs were rescaled so that the operating threshold corresponded to 0.50 on the dashboard, providing a consistent boundary for displaying higher-risk results. Risk estimates were updated every 15 min. In the test dataset, the model achieved a sensitivity of 0.858 and specificity of 0.858, with an overall AUC of 0.936. Additional threshold-dependent performance or alert-burden measures may be specified where clinically relevant to the intended use and workflow. In the Explainability domain, clinicians are provided with clinically relevant context, including blood-pressure trends, ultrafiltration information, prior-session data, and SHAP-based feature attribution, together with appropriate disclosure of uncertainty and the non-causal nature of feature contributions. In the Communication domain, risk is presented through stratified displays and alert messages linked to relevant standard operating procedures (SOPs). Acknowledgement and override mechanisms allow clinicians to retain decision authority, while action records support traceability and audit.

The deployed GAI service provides a practical context for specifying governance requirements across the three domains. In the Capability domain, specifications should define the permitted support tasks, output constraints, model and prompt versions, and conditions for withholding generation or requesting human review when source information is insufficient. In the Explainability domain, specifications should identify the source information needed to verify generated content and distinguish that information from model-generated synthesis. In the Communication domain, any incorporation of generated content into the clinical record should require review and confirmation by an authorized clinician, with explicit write-back restrictions and retention of relevant source information and user modifications. These are application-level governance specifications illustrated through the service, rather than a claim that every listed control has been fully implemented or evaluated.

These two applications demonstrate why a shared governance vocabulary is useful without implying that PAI and GAI require identical controls. The same three domains identify where governance must become operational, but the required specifications depend on the nature of the AI output: probabilistic risk prediction emphasizes calibration, thresholds, uncertainty, and alert handling, whereas generative support emphasizes task boundaries, provenance, human verification, and controlled incorporation of generated content into the clinical record.

## 6 Implications for healthcare institutions

This reflects the broader socio-technical view that trustworthy AI depends not only on model properties but also on workflow integration, human–AI role delineation, accountability, failure visibility, and continuous evaluation (24). The principle-to-practice gap emerges when institutional requirements are not translated into these application-level controls.

Using Capability, Explainability, and Communication as functional governance domains can support a more structured review process. An AI governance committee can ask not only whether a system is “transparent” or “human supervised,” but where transparency is implemented, which limitations are visible, which user is expected to review the output, what action is permitted, and which monitoring indicator will reveal deterioration or misuse. The same vocabulary can be used by clinical teams, information departments, AI centers, cybersecurity teams, and governance reviewers, while the required controls remain proportional to clinical risk.

This perspective also clarifies the relationship between governance and system design. Governance should not be applied only after an AI service is built. Application-level specifications can function prospectively as design requirements and retrospectively as review criteria. In practice, the relationship is iterative: work-as-imagined in governance documents must be reconciled with work-as-done in clinical settings. Differences between intended and actual users, alert handling, review behavior, or documentation practices should trigger redesign rather than simply additional policy.

## 7 Discussion and future directions

The central argument of this Perspective is that responsible clinical AI governance becomes actionable when principles are attached to identifiable functional points in the service. Capability, Explainability, and Communication provide a pragmatic organizing structure because they correspond to recurring questions about output definition, user understanding, and clinical use. Together, these domains provide an operational bridge between high-level responsible AI principles and the specific controls required when predictive and generative AI are implemented in clinical practice.

This framing has limitations. The proposed specifications are derived from established governance guidance, clinical AI literature, and practical implementation experience, but they are not exhaustive and have not been validated as a universal instrument. Different specialties, risk levels, user roles, and organizational maturities may require additional controls. The two dialysis applications are illustrative rather than comparative evaluations, and they should not be interpreted as evidence that the three-domain approach improves safety, usability, or clinical outcomes.

Future work should evaluate whether domain-based specifications improve the consistency and completeness of AI governance review across applications and institutions. Candidate approaches include expert assessment of relevance and feasibility, structured clinical walkthroughs, cross-institutional comparison, and prospective measurement of operational indicators such as alert burden, override patterns, review time, adoption, revision of generated drafts, and safety events. For GAI in particular, further work is needed to define clinically meaningful measures of traceability, refusal behavior, human verification, and over-reliance. Future evaluation should also examine which application-level controls are essential across settings and which should vary according to clinical risk and organizational context.

As PAI and GAI increasingly coexist in healthcare, governance should neither collapse them into a single model-risk category nor create entirely separate governance systems. A shared set of functional domains with differentiated specifications for PAI and GAI offers a middle path. It enables healthcare institutions to maintain common governance language while recognizing that probabilistic prediction and generative content require different evidence, disclosures, monitoring, and human-use controls.

## 8 Conclusion

Responsible AI governance in healthcare must move from broad principles to controls that are visible and enforceable at the level of individual AI services. Capability, Explainability, and Communication can serve as functional domains for organizing these controls across both predictive and generative AI. Importantly, these application-level specifications can serve prospectively as design and pre-deployment review requirements and retrospectively as criteria for monitoring, audit, and change control after deployment. As models, workflows, and clinical contexts evolve, governance should therefore assess not only whether an AI service continues to perform adequately, but also whether its capability boundaries, verification requirements, and workflow authority remain appropriate for its intended clinical use. This lifecycle perspective positions responsible AI governance as an ongoing process rather than a one-time approval decision, supporting continuing oversight of AI services in clinical practice.
